# New Jersey State Dental Society

**Published:** 1889-10

**Authors:** 


### NEW JERSEY STATE DENTAL SOCIETY.—(*Continued.*)

### DISCUSSION ON DR. R. C. NEWTON'S PAPER ENTITLED "THE TEETH AS A FACTOR IN DIAGNOSIS."

*Dr. James Truman.*—Mr. Chairman, I was very much interested in the paper, from the beginning to the end. There are many points in it that should require careful consideration, I think, and yet I do not agree with some of the statements, which I regard as not being absolutely proven. The idea that syphilis invariably produces a certain class of teeth seems to me an erroneous one. I know very well that we have the idea of Hutchinson, of peg teeth, and, as Dr. Foster has said, that notched teeth are an indication of syphilis. I do not agree with him. It is very evident to my mind that teeth of the character mentioned are found where there certainly had not been syphilis. I have patients with peg teeth in whose cases there was no pathological condition preceding the development of those teeth that could have produced them; and I know of no reason why this character of teeth should be ascribed to certain syphilitic conditions, or to rickets.

In the matter of idiots' teeth I agree with Dr. Foster, that a saddle-shaped jaw is no indication of idiocy. The conclusions in this matter are mainly derived, I think, from mistaken observation. Persons will go to the different institutions of the country and examine the teeth of all the children they find there, without regard to former conditions, whether they are congenital or otherwise. During my connection with the institution for the blind in Philadelphia, I found that a large proportion of the children acquired their blindness through accident or disease, and that congenital cases were comparatively rare; also, that the teeth of the different children were about equal, so far as irregularity and density were concerned. I am very sceptical in regard to examinations of this character; I do not believe they lead to correct conclusions.

In regard to mouth-breathing as a source of irregularity, it seems to me to be a positive error. There are many persons who breathe only in that way day and night, and I imagine that we cannot find a great amount of irregularity from this cause. Sucking of the thumbs might produce it, but it is difficult to imagine how the pressure of the muscles on the teeth in mouth-breathing can produce the disturbance complained of. Irregularities come from other sources, and these we all understand.

Again, I do not hold to the idea that Americans are so badly off for teeth. The charge has been sounded for many years in our medical associations, and it has been carried abroad, until the American is looked upon as having the poorest teeth. My experience—and it is quite an extensive one, having lived abroad for some time—has taught me that the American teeth are, as a rule, quite up to the average standard, or at least are coming to that. The typical form of American teeth is about as good, taking the average, as any I have seen. We have descended from English, Irish, and German stock; and there has been a deterioration in our teeth, from climatic influences and other causes, and there must be a return to the original type, because all nature seeks an equilibrium and comes back to the original character sooner or later. We are reaching that point, and largely, I think, through the efforts of the profession of which I am pleased to be a member. While bad air and poor conditions produce injurious results, as we all know, I have nevertheless seen as good teeth from children that have been raised in bad air as are found in better conditions. I have examined in Germany the teeth of children dwelling in cellars, where there was naturally a great scarcity of anything like a healthy atmosphere, and I failed to discover any marked difference in the character of the teeth from those more favorably situated. Bright's disease and gouty diathesis have in my opinion a local effect. All those diseases produce a more or less acid reaction in the secretions, and that reaction produces local disturbance, and I do not believe they have any direct effect systemically. The same thing occurs in loss of teeth from shock. All conditions of that kind produce a change in the secretions, and we have an acid action upon the teeth. The question is a very large one, and I do not feel like going into it very extensively, as time will not permit.

*Dr. Peirce.*—Mr. President, I agree with Dr. Truman, that this subject is a broad and very important one, and that any one who speaks upon it needs to weigh his words carefully, or he will find himself advancing ideas that he cannot sustain. The whole paper was one of great interest; it goes over ground with which I have been somewhat familiar. Taking it up in the order adopted by the author, the first item to which he referred was syphilitic teeth and the influence of syphilis in their premature development or eruption, as it is termed. Where teeth are prematurely erupted, as at birth or soon thereafter, the organs themselves do not vary in their development from a normal condition; there is simply a waste of superimposed tissue, and they are prematurely erupted by reason of this early absorption of the soft structure which covers their crowns. This occurs where we have ill health or improper nutrition from any cause. A tooth erupted at birth has only a membranous connection or attachment; it has no developed root, and corresponds exactly to the normal tooth; that is, the crown is the same in its calcification, but there is no root; and so in the tooth that is erupted, two months after birth we have calcification of the crown and a slight calcification of the neck, same as normal.

As to the forms of syphilitic teeth, I have had several patients—children—who were evidently suffering from this systemic disturbance, manifesting itself not in the teeth, but in the bones, as an inheritance from the parents. The teeth of these children were well developed. As an example, a lad of twelve years, suffering from maxillary necrosis, with every tooth in the mouth in excellent condition, so far as formation and freedom from decay were concerned; but the inferior maxilla, from the ramus on one side to the bicuspids on the other, had died, and during or within a period of six or seven months the teeth were necessarily lost with the bone. To repeat, all that part of the inferior maxilla, from and including the ramus on the left side to the second bicuspid on the right, was lost with the teeth located thereon, which were well formed. When the mother was asked regarding her condition during and previous to gestation, she said her husband was in the army, and that he came therefrom diseased, and that she suffered afterwards from like condition. The child had been ordinarily healthy until twelve years of age, when, being subjected to inclement weather, he contracted a cold, and inflammation set in, with the above described result. It was certainly attributable to the previous condition of the parents.

The teeth described by Hutchinson have often been misrepresented by physicians, because they have not accurately studied the markings resulting from the disease. In the superior jaw he represents teeth blunt and concave at the cutting edge; in the inferior jaw they have constricted necks and are concave at the cutting edge. Whether that is induced by improper nutrition or from other considerations, I am not prepared to say; but that it is always present where children suffer from hereditary syphilis is not consistent with my experience. That we find conditions similar to it, where there has been disease during the early periods of the development of the germs of the teeth, and in their progressive stages, I am well satisfied. We find teeth with restricted necks and with pits and rough edges; but I do not think I ever saw any with the peculiar peg and concave form, as described by Hutchinson, from any other condition than syphilitic taint. The deciduous teeth are usually free from these imperfections, because they have progressed in their development to the calcification of the crowns before birth. The calcification of the permanent teeth commencing at birth, their imperfections may be due to disease or any condition that may induce mal-nutrition. The extent of these imperfections will depend upon the duration and severity of the abnormality. If this systemic disturbance occurs during the first year, the ill development will be on or near the cutting edges; if a year later, it will be farther up on the crown and on all the teeth developing at that time. If it occurs in the fourth year, the incisors would be free from it and the second molars would be somewhat affected, indicating that the imperfections have their origin in systemic abnormality, their location corresponding with the age and progressive development of the tooth or teeth. I have never noticed imperfections in teeth that I could not attribute to some interruption of nutrition during the period in which those teeth were being calcified.

Dr. Allen's name has been mentioned in the paper. I have been interested in sending him some patients in whom gouty teeth were well marked. I have observed that the effect of this disease upon teeth was, as in syphilis, not uniform. The tooth that has been figured as a gouty tooth is one that is thick and short,—that is, the congenital gouty tooth is yellow, short and thick; and teeth that have been used to indicate gouty diathesis are abnormally developed in the manner described. Oftentimes we see children of gouty parents in whom the results of that disease upon the teeth are not developed until late in life; the teeth have been normal in form and development; but in subsequent years they have shown the markings and abrasions on the labial surfaces and the cutting edges which we have believed to result from a gouty diathesis. I have in my practice a large family, one of whom is to-day a great sufferer from gout; her teeth are not affected, but her joints are so enlarged that she can move only with great discomfort. Another sister has her teeth cut so on the labial surfaces that the enamel is almost removed; very little, indeed, remains upon the labial surfaces of the incisors and cuspids. The molars are not so affected.

*Dr. Foster.*—Are they irregularly marked, or abraded?

*Dr. Peirce.*—They have a crescent-shaped abrasion, covering about two-thirds of the surface from the neck towards the cutting edge. Of the father I have not definite knowledge; he died in middle life. The mother is living, and is apparently a healthy woman. In this family you see a great difference; one, a great sufferer, has good teeth, free from any gouty marks; and another, able to work at her profession, has teeth marked prominently as I have described.

Of the teeth producing facial neuralgia, the author of the paper makes mention. The influence of various abnormal conditions of the teeth in producing facial neuralgia cannot be ignored, indeed is fully recognized by every dentist. There are many persons under the care of physicians who suffer for months, or it may be years, with this distressing expression of abnormality; the physician after exhausting his pharmacopœia in trying to relieve the discomfort, sends his patient to the dentist. In this specialist's hands the teeth are found sound, so far as decay is concerned, but dental excementosis inducing pericementitis, or pulp nodules inducing pulpitis, may be found there. Either of these conditions is a prolific source of facial neuralgia.

The inquiry has been made as to whether accumulations upon the teeth are indications of renal or hepatic deposits. I should say only as indicating a predisposition to lime-deposits, or the excess of this salt in the blood.

As to the tubercular teeth of children, the influence of phthisis upon children's teeth depends upon the extent and condition of the disease. In the case of a child whose mother suffered with phthisis during the whole period of gestation, the deciduous teeth were erupted without enamel. They remained the usual time, were shed, and the permanent teeth were erupted with an imperfect enamel covering also. The child was never well, though it lived to be fourteen years of age. The imperfect development was due to improper nutrition; and I would not like to say that there was any special marking upon the teeth as indicative of a tuberculous diathesis.

With reference to the teeth of idiots, my experience has been that there is an arrest of development, not only of brain and brain-force, but of the jaws and teeth,—a reversion to an ancestral type in appearance,—the jaws large, and the teeth arranged in a straggling manner, with spaces between them.

Aurists have long recognized the teeth as important factors in disturbances under their care, and every observing dentist recognizes the fact that an exposed pulp or an excementosed root will produce pain in the eye or in the ear; and the reason for it is very easily explained on the theory of reflex influence.

In regard to mouth-breathing, this is a subject of much interest. In cases of congenitally-enlarged tonsils the child will necessarily sleep with its mouth open, the act of breathing necessitating this. The positions of the deciduous teeth are not affected by it, for the reason that they develop early in life and before the muscles become rigid; but if the enlargement of the tonsils continues, and consequently the mouth-breathing, the constant pressure of the muscles upon the sides of the jaws, as the teeth are gradually being evolved, results in a narrow jaw and protruding front teeth. The first variation is enlargement of the tonsils; the next, and concomitant, is contraction laterally with an accompanying deep and high arch. The tension of the muscles of the cheek and pressure upon the bicuspids must necessarily narrow the arch and protrude the anterior teeth.

The influence of a soft tumor upon the teeth is well recognized by every dentist, and in the same manner the tension of the muscles will displace the teeth.

I think that one of the chief causes of defective teeth in many misses is excessive mental effort. Certainly every dentist recognizes the difficulty he experiences from the deleterious influence upon the teeth of girls who strive for prizes in the schools. In the three or four months before the end of the school term, when the girls are making great mental efforts to obtain the prizes, there is a very marked change in the condition of their teeth; nutrition is diverted and the nervous system placed in an irritable condition, and the teeth suffer. The teeth of misses have given me more trouble during this period of their school terms than those of all my other patients.

*Dr. Newton.*—How is it with boys?

*Dr. Peirce.*—Boys will not give their whole attention to study; they divert themselves with base-ball, and it is a good thing for them. This modification in tooth-structure and development may be illustrated from comparative biology and physiology.

As we rise in the scale of intelligence we get a shorter jaw and fewer teeth. Even the horse of to-day has four teeth less than the horse of a few centuries ago. The race-horse has lost in the size of his jaw and the number of his teeth. That is caused by diversion of nutrition. This is a physiological and biological fact, and upon that fact some of our comparative anatomists and naturalists have predicted that man will eventually become edentulous.

*Dr. Watkins.*—Dr. Truman stated that he did not believe mouth-breathing caused irregularity. I have a case in my mind now, of a young girl whom I have seen constantly from infancy; at the age of five years she was affected with St. Vitus's dance, and went around with her mouth open, the under jaw hanging, and the saliva running out of her mouth. She was in that condition for about three years, and ever since she has breathed through her mouth. At the beginning of that trouble she had a good-shaped jaw and regular temporary teeth. The jaw began to contract, and continued till, at the age of eleven years, the upper jaw was so narrow and the teeth so close together that you could not place the tip of your first finger between them all the way up in the roof of the mouth; the temporary lateral incisors were entirely covered by the centrals, and the cuspids lapped over the edges of the centrals; the bicuspids were standing in and out of the arch, and there was about space for ten teeth instead of fourteen.

I had another case, of a boy, who had a good round arch in infancy. At about the age of three years he had his nose broken. It was neglected at the time, and one nostril partly closed, so that he breathed with difficulty. The result was mouth-breathing; and the consequent pressure of the muscles of the cheek upon the temporary molars has so contracted the arch that now, at the age of ten, there is not much more than space enough to put your finger between the teeth in the roof of the mouth. The arch, which was round, has become deep and narrow. I have seen both cases from the infancy of the patients, and I cannot ascribe the irregularities to any other cause than mouth-breathing.

*Dr. Atkinson.*—We are in deep water. I have heard enough this morning, in the reading of the paper and the discussion of it, to lay the foundation of a very good syllabus, pointing towards a solution of this question; but as to the solution of the question, there is not coherence enough in the concensus of the minds investigating it, and the terminology used, to enable us to correlate what has been said into an understandable and useful shape, that we can carry home with us and apply in anything like regular order of succession in understanding the conditions and knowing how to meet the demands. There is one man who has given more attention to this question than any other man within the range of my knowledge. He was in charge of the effete nobility of France, who had by intermarriage become so afflicted with distorted features as to make it pretty certain that if you met a very ugly woman or man on the streets of Paris, that person was a member of the nobility. Without much erudition or knowledge, he was able to get control of those people, and by means of mechanical appliances he has brought back their features to a presentable shape. If this is true, it has much to do with our inquiry as to where the deflection from the normal standard takes place; how any dereliction is always tending towards bad development.

If we would study the development of the organs in question from the time of the differentiation of Meckel's cartilage all the way through wherever histology would lead us, and we had the time,—not having to fight like beavers for our daily subsistence,—we might arrive at something profitable to talk about. And I am only talking now. I wish there was nothing to do but that kind of talking by question and answer which brings out the ideas of different minds; and then, if men were sufficiently in earnest to receive some kind of impression from what they have heard, we would travel more together and be able to appreciate in some measure this subject, which is incomprehensible without the deep understanding of the laws of primogeniture and heredity; and how it is that past molecular experiences of antecedents are stored in the organs that we have to deal with, causing them to be built according to the typical standard of organs; these principles, I say, will have to be better studied than they have been before we make much progress. I notice that every clean-cut, bright-minded physician or surgeon, who goes about his work with an earnestness that is not perfunctory, who feels the responsibility of his office and tries to get a good mental grip of his subject to enable him to benefit his patients, will hesitate about making prescriptions. But the many seem to make an effort to get rid of the responsibility by writing some cabalistic sort of necromancy on paper and sending it to an apothecary. When we are weak enough for this sort of effeminacy we would better confine ourselves to mechanical appliances.

*Dr. Rhein.*—Mr. President, the subject of the paper, "The Teeth as a Factor in Diagnosis," has a peculiar interest for me, because for the past two years I have been making investigations that tend to show the teeth to be a very important factor in aiding the physician in diagnosing a number of constitutional disorders and systemic troubles at the commencement of those troubles, before they assume a well-defined shape and are not clear to the eye. The results that I have already obtained have been sufficient to enable me to speak quite positively in opposition to the views advanced by Professor Truman on this subject. The disease that has been particularly engrossing my attention in this direction is pyorrhœa alveolaris, in cases where it has been demonstrated that the affection is the result of some general systemic disturbance. In investigating this subject I have met with several cases where there was no evidence whatever of any apparent local disturbance, such as a decided acid reaction, the oral secretions being apparently in a healthy condition, the teeth good, and the mouth in the cleanliest condition; and yet at intervals the patients suffer the severest pain, there would be an œdematous condition of the soft tissues above the necks of the teeth, resulting, if neglected, in a discharge of pus from the sockets of the teeth, but which would disappear upon the patient following out a system of prescribed hygiene. In these cases an exploring instrument passed under the gum soon reaches the pocket or pouch which we find in all forms of pyorrhoea alveolaris, and which arises from lack of nutrition in the pericemental membrane. To my mind it has been positively proved that it arises right in that region from mal-nutrition, or lack of nutrition, resulting, in extreme cases, in a retrograde metamorphosis of the tissues, and partial destruction of the roots of the teeth. I have in my office a tooth the root of which has been half absorbed by pyorrhœa alveolaris, presenting a condition similar to some implantation failures. One point of difference that has particularly attracted my attention is that in the absorption of the root, after a tooth has been implanted, or replanted, the edges are left very sharp and well defined, while in cases of retrograde metamorphosis due to systemic disturbance the edges are quite rounded and oval, not having that saw-like appearance possessed by the others. I desire particularly to bring up that one point in contradistinction to the remarks made by Professor Truman as to all those forms being due to local disturbance of the parts. And I wanted to add a word to the remarks of Professor Pierce on trigeminal neuralgia, where there was no evidence of dental caries, nor any evidence of any kind of trouble about the teeth. He limits the cause, if it does arise from the teeth, to exostosis and to pulp nodules. I want to add a cause which I have found almost as frequently as the others,—that is, an embeded tooth or root.

*Dr. Atkinson.*—I failed entirely to mention one most important point, in my estimation,—the mistake which every speaker that I have noticed has made in regard to the premature eruption of deciduous teeth; they misapprehend the actual condition, and talk of the soft parts over the erupted tooth as having been absorbed. They do not remember, or else never knew, that that tooth never had a calcified tissue over it; it has nothing but connective tissue-fibres over it to hold the crown, generally an incisor, either central or lateral. And then it was said that they never had any roots. How do they know that?

*Dr. Pierce.*—The doctor wants to know whether I have any knowledge that the teeth erupted at birth have no roots. I have knowledge, because I have taken them out and found they had no roots.

*Dr. Atkinson.*—If anybody else but a real morphologist had said that, I would have said it did not demand a reply; it is so puerile. They had no roots; of course they had not. But how do you know they would not have had roots if you had allowed them to remain the proper time? Why consume our time with such figments of knowledge! Had he let them alone he would have been able to determine, at the adult period of the child, whether they did have a root or not. I have seen them that did have roots, and remained there. I have taken such teeth out, too, just for the reason that I loved the mother and did not want to have her blessed fountain of pabulum interfered with by the irritation of that little thing that came out of time and place.

Here we are stumbling about what absorption is, and the removal of deciduous tissues. Do not let us make the mistake of thinking that every tooth must finally be like every other tooth, and that the multi-cuspid teeth must have multi-roots,—many roots. I do not claim to have been in the counsels of the infinite producer of all these things to hear how he has formulated them exactly, but I have smelled through the keyhole, and have learned a few of the types, so that I am somewhat familiar with the subject. All these bodies are loaded down by being laid together as antecedents of the tissues. We say dentine comes from the dentinoblasts. They have been called odontoblast. Call them what you like; they are little bodies that are capable of holding the stored radiancy that is to form that type of tooth, so as to appropriate the lime-salts, and become hard enough to perform its function. We want to know something about the antecedent potency that determines whether there shall be one, two, or three roots to teeth.

*Dr. James Truman.*—I have been called to task in regard to my views of mouth-breathing. I would like to know how it is possible to produce that contraction of the jaw by simply breathing. There is certainly no pressure against the teeth from keeping the mouth open. Any individual in this room can breathe with the mouth open and not move the muscles at all. There is not enough pressure of the muscles upon the teeth to produce any irregularity. A member of my family, owing to a short upper lip, breathed that way all through life, and had a perfectly regular set of teeth.

*Dr. Newton.*—I am exceedingly obliged to the gentlemen who have followed me; the discussion has been extremely interesting and instructive to me. I came here to learn, not to teach anybody.

There has been nothing said, that I remember, in this discussion about Bright's disease. That is a point that every general practitioner is deeply interested in, and covers an immense amount of ground, particularly the sensitiveness, or hyper-sensitiveness of the nerves in that disease. If hyper-sensitiveness of the teeth is a symptom of Bright's disease, either constant or occasional, we want to know it. It was a very strange thing to me, when my friend Dr. Watkins told me he had noticed that condition in Bright's disease. I do not know that it has any connection with the disease, but we must not jump at conclusions. If you gentlemen will bear this in mind, and study the condition of the teeth in Bright's disease, and report your observations, you will be conferring a benefit upon humanity. I am very much obliged to you all for the kindly spirit in which you have received my paper.

On motion, the paper was passed.

(To be continued.)

